# Assessment of Efficacy and Safety Using PPAR-γ Agonist-Loaded Nanocarriers for Inflammatory Eye Diseases

**DOI:** 10.3390/ijms231911184

**Published:** 2022-09-23

**Authors:** Esther Miralles, Christina S. Kamma-Lorger, Òscar Domènech, Lilian Sosa, Isidre Casals, Ana Cristina Calpena, Marcelle Silva-Abreu

**Affiliations:** 1 CCiTUB (Scientific and Technological Centers), University of Barcelona, 08028 Barcelona, Spain; 2 Scattering Group, ANSTO Australian Synchrotron, Clayton, VIC 3168, Australia; 3 Department of Pharmacy, Pharmaceutical Technology and Physical Chemistry, Faculty of Pharmacy and Food Sciences, University of Barcelona, 08028 Barcelona, Spain; 4 Institute of Nanoscience and Nanotechnology (IN2UB), University of Barcelona, 08028 Barcelona, Spain; 5 Pharmaceutical Technology Research Group, Faculty of Chemical Sciences and Pharmacy, National Autonomous University of Honduras (UNAH), Tegucigalpa 11101, Honduras

**Keywords:** PPAR-γ agonist, polymeric nanocarriers, eye inflammation, ocular efficacy, corneal tissue

## Abstract

Drug-loaded nanocarriers (NCs) are new systems that can greatly improve the delivery and targeting of drugs to specific tissues and organs. In our work, a PPAR-γ agonist loaded into polymeric NCs was prepared, stabilized by spray-drying, and tested in vitro, ex vivo, and in vivo (animal models) to provide a safe formulation for optical anti-inflammatory treatments. The NCs were shown to be well tolerated, and no signs of irritancy or alterations of the eye properties were detected by the in vitro HET-CAM test and in vivo Draize test. Furthermore, no signs of cytotoxicity were found in the NC formulations on retinoblastoma cells (Y-79) analyzed using the alamarBlue assay, and the transmittance experiments evidenced good corneal transparency with the formulations tested. The ocular anti-inflammatory study confirmed the significant prevention efficacy using the NCs, and these systems did not affect the corneal tissue structure. Moreover, the animal corneal structure treated with the NCs was analyzed using X-ray diffraction using synchrotron light. Small-angle X-ray scattering (SAXS) analysis did not show a significant difference in corneal collagen interfibrillar spacing after the treatment with freshly prepared NCs or NCs after the drying process compared to the corresponding negative control when inflammation was induced. Considering these results, the PPAR-γ agonist NCs could be a safe and effective alternative for the treatment of inflammatory ocular processes.

## 1. Introduction

The human cornea consists of five layers: the epithelium, Bowman’s layer, stroma, Descemet’s layer, and endothelium, with the stroma constituting about 90% of the thickness of the cornea. The stroma consists mainly of collagen and cells, and it is responsible for the transparency of the cornea. However, in 2013, a sixth layer was discovered, which was named Dua’s layer [1]. This layer is a well-defined, tough acellular lining, only 10 μm to 15 μm thick, sandwiched between the corneal stroma and Descemet’s membrane [2].

If the ordered collagen structure of the cornea is disrupted, such as in the case of inflammation, caused by injury or disease, the cornea becomes opaque, and the normal visual function is put at risk. Therefore, it is very important to find medical agents that are capable of recovering the normal structure of the cornea [3,4].

The mechanical strength of the cornea, together with its refractive index and transparency, is directly related to the collagen arrangement in its lamellar structure, which contains long collagen fibrils of uniform diameter. Small-angle X-ray scattering (SAXS) and wide-angle X-ray scattering (WAXS) techniques have been widely used to study collagen ultrastructure in the cornea and to observe differences between healthy and diseased tissue. They have been proven valuable tools in evaluating structural changes in the tissue [5]. SAXS allows the measurement of the average fibril center-to-center spacing (interfibrillar spacing) and fibril diameter [6]. WAXS allows the calculation of the collagen intermolecular spacing and the orientation of corneal collagen fibrils [7,8,9].

Pioglitazone (PZ) is a drug used to treat type 2 diabetes by enhancing insulin sensitivity [10], acting as an agonist of peroxisome proliferator-activated receptor gamma (PPAR-γ), a nuclear receptor that regulates glucose homeostasis, lipid metabolism, and inflammation [11]. Therefore, PZ has been investigated beyond its primary use, and studies are looking into it as an anti-inflammatory, anti-arteriosclerotic, antifibrotic, or neovascularization agent [12,13,14,15,16].

This drug belongs to class II in the Biopharmaceutic Classification System (BCS) [17], which has poor aqueous solubility and high permeability. These factors could reduce the absorption of the drug and decrease the dissolution rate, adversely affecting the levels of the drug in blood and consequently decreasing the pharmacological activity. Different approaches have been explored to deal with the limitations of low water solubility to improve its transport [18,19,20,21,22].

Polymeric nanocarriers (NCs) have been developed to improve drug targeting to tissues and organs and to increase drug bioavailability across biological membranes. Poly(lactic-*co*-glycolic) acid (PLGA) is a polymer approved by the FDA for its use in humans [23], and it is the most widely used vehicle in drug delivery research and therapeutic devices, due to its biodegradability and biocompatibility. PLGA must be able to deliver its payload with appropriate duration, biodistribution, and concentration for the intended therapeutic effect, loading hydrophilic and hydrophobic molecules. To promote the stability of the nanomaterials and increase the circulation time because of the ‘stealth’ effect on the macrophages, polyethylene glycol (PEG) is used in association with PLGA [24,25,26].

Polymeric NCs are obtained mainly as aqueous suspensions, which present stability problems during their storage, such as aggregation, hydrolysis, or drug leakage. Spray-drying technology is an alternative used for the rapid elimination of water in the fields of chemical materials, cosmetics, food, and flavor, as well as the pharmaceutical industry [27], at both laboratory and industrial scale. The dry powder obtained in dehydrating nanosuspensions, before being resuspended for use, must maintain the physicochemical properties and efficacy.

Classical ocular drug delivery systems often fail to combat diseases due to poor ocular bioavailability and low ocular residence time. Therefore, the use of polymeric nanosystems has generated interest for ophthalmic drug delivery. Recently published reviews [28,29,30] concerning the use of nanotherapeutic systems for the treatment of ocular diseases demonstrated that these systems improve the efficacy of drugs by increasing their penetration into the cornea. Usually, in clinical practice, ocular inflammatory disorders are treated with corticosteroids, and the field of nanoparticles is being explored as there is a need to enhance the bioavailability of these drugs [31,32]. In addition, the encapsulation of nonsteroidal anti-inflammatory drugs (NSAIDs) into nanoparticles for ocular route delivery has been investigated [33,34]. Topical application of drug solutions (eye drops) is the preferred route of administration for the treatment of eye disorders [35]. These conventional dosage forms must be sterile, stable, and isotonic, and they must not create irritation or vision problems.

In previous studies, PLGA-PEG-PZ NCs were formulated, optimized and characterized physiochemically, and stabilized using the spray-drying technique [36] in order to be used in ocular diseases, but no studies have been conducted to assess the performance and safety of these NCs. A variety of techniques were used in the present study, including tolerability assays, cell toxicity, in vivo anti-inflammation assays, histological visualization, synchrotron SAXS analysis, and corneal transparency, to determine the ocular safety of these systems.

## 2. Results and Discussion

### 2.1. Physicochemical Characterization of PZ-NCs

In our previous studies, PZ-NCs of PLGA-PEG were optimized and characterized physiochemically and biopharmaceutically as a new delivery system suitable for the ocular route [36,37]. PZ-NCs were previously synthesized using the solvent displacement technique, showing a particle size of 247.22 ± 2.77 nm with polydispersity index (PI) values lower than 0.2 (0.17 ± 0.03) and a negative charge with zeta potential (ZP) values of −3.34 ± 0.42 mV. Moreover, the percentage of PZ encapsulated (EE) was 90.12% ± 1.15%. To stabilize the PZ-NCs, a spray-drying process was carried out. After that, the NCs were resuspended in water, and the values found were a particle size of 271.31 ± 2.78 nm, PI of approximately 0.24 ± 0.00, ZP of −7.57 ± 1.53 mV, and EE of 89.13% ± 2.21%. These physiochemical parameters before and after the spray-drying process were considered suitable for ocular delivery [36,37]. These parameters are in concordance with other authors for the use of polymeric systems in ocular therapies [18,28,38,39].

### 2.2. In Vitro HET-CAM Test

In order to evaluate the ocular tolerability of the developed formulation, the hen’s egg test with a chorioallantoic membrane (HET-CAM) was carried out. The potential irritancy of compounds may be detected by observing adverse changes that occur in the CAM of the egg after exposure to test chemicals [40]. The HET-CAM test is based on the direct application of the sample into the chorioallantoic membrane and the observation of reactions such as hemorrhage, intravasal coagulation, or lysis of blood vessels. The results of the assay showed that the positive control (NaOH 0.1 M) caused severe hemorrhage, lysis, and coagulation, increasing after 5 min to become a strongly irritating solution. However, the NC formulations did not irritate the chorioallantoic membrane within 5 min of the assay (score 0), thus revealing optimal ocular tolerance (Figure S1).

### 2.3. In Vivo Ocular Tolerance (Draize Test)

The in vivo Draize test [41] was performed to confirm the results obtained by the HET-CAM assay. In the present work, the irritancy of the developed PZ-NCs formulations was evaluated in New Zealand white rabbits. No signs of ocular irritancy or damage were detected after PZ-NCs instillation at the studied times, with zero scores in all cases for both formulations, i.e., freshly prepared or resuspended after spray-drying (image not shown). Moreover, these results agree with previous studies developed using PLGA-NCs [42,43] and also are in concordance with the HET-CAM test confirming the nonirritant potential of PZ-loaded polymeric nanoparticles [42,43].

### 2.4. In Vivo Efficacy Assay: Anti-Inflammatory and Histological Studies

**Figure 1:**
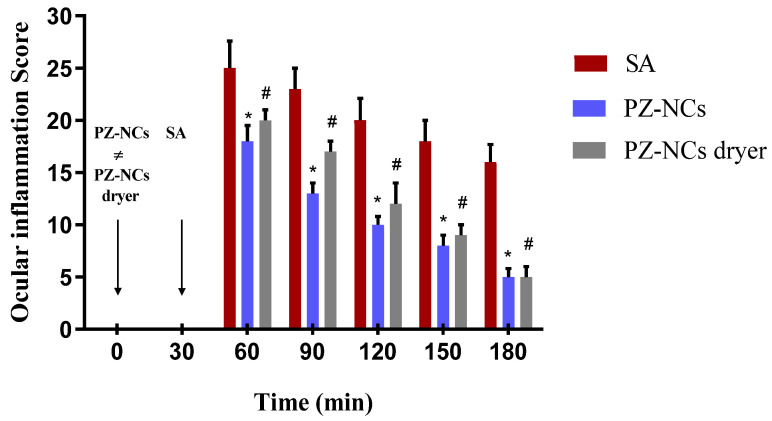
Comparison of prevention anti-inflammatory efficacy after sodium arachidonate (SA)-induced inflammation in the rabbit’s eye. Values are expressed as the average ± SD (*n* = 3); * *p* < 0.05 comparison of PZ-NCs vs. positive control (SA); ^#^ *p* < 0.05 comparison of PZ-NCs dryer vs. positive control (SA).

**Figure 2:**
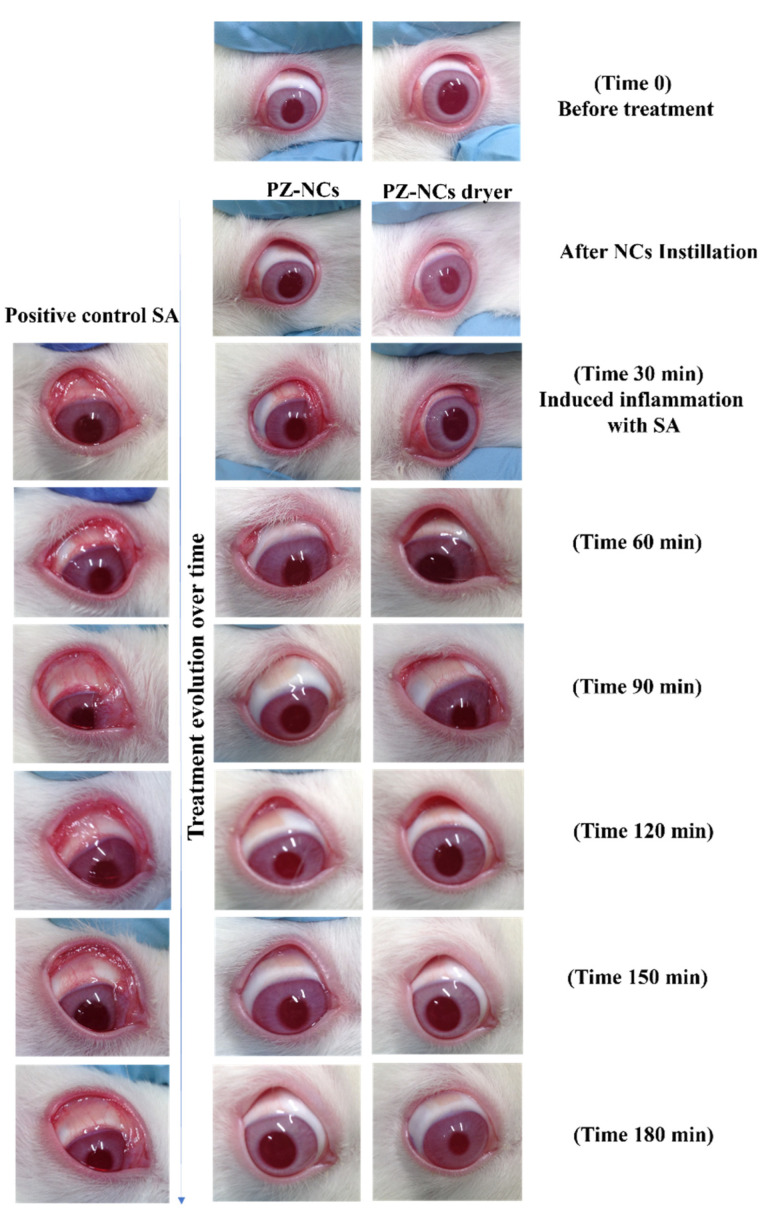
Anti-inflammatory efficacy of different formulations containing PZ in the ocular edema induced by instillation of sodium arachidonate (SA). Time 0 = negative control (without treatment).

One method of evaluating the ocular anti-inflammatory efficacy of PZ-NCs formulations consists of administering the samples to rabbits 30 min before inducing inflammation with sodium arachidonate (SA). In this study, the PZ-NC formulations showed significant differences in the prevention profile in comparison to the positive control at all times tested (*p* < 0.05) with a higher decrease in scores over time (Figure 1). They significantly reduced the degree of conjunctival inflammation and iris hyperemia (Figure 2). Moreover, the polymer used to prepare the NCs was PLGA-PEG, which affects the ocular bioavailability of drugs due to its special behavior that facilitates drug–mucin interactions, including mucoadhesive and mucus-penetrating properties [44,45,46,47,48]. According to another study, mucoadhesivity in the PLGA-PEG NCs can be related to the PEG corona as it allows a better and longer interaction between the particles and the eye [49]. Furthermore, the PEG surface increases nanoparticle permeability and influences the anti-inflammatory effect when compared with formulations with only PLGA [48,50]. This result fits in with that of other authors regarding the efficacy of using these systems [51,52,53].

**Figure 3:**
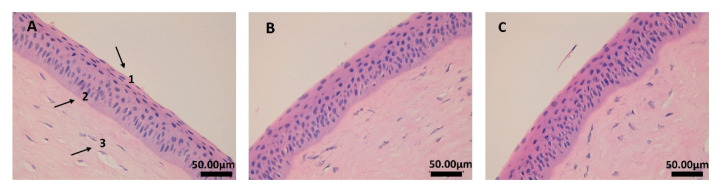
Corneal histological structure: (**A**) negative control (cornea without treatment); (**B**) cornea treated with PZ-NCs; (**C**) cornea treated with PZ-NCs dryer. 1: Stratified flat keratinized epithelium; 2: Bowman’s membrane; 3: own laminate. All images were observed at 400×.

Regarding histological studies, it is possible to observe the corneal structure in Figure 3. The pictures confirm that the tissue and structure were preserved using both formulations of NCs. The epithelium (1) and the own laminate (3) are separated by a thin layer called the Bowman membrane (2). As can be seen in images B and C, the corneal structure was not affected, and the epithelium (1) cells were not altered by any of the formulations tested.

### 2.5. Toxicity Assay (Cell Culture Cell Line Y-79)

**Figure 4:**
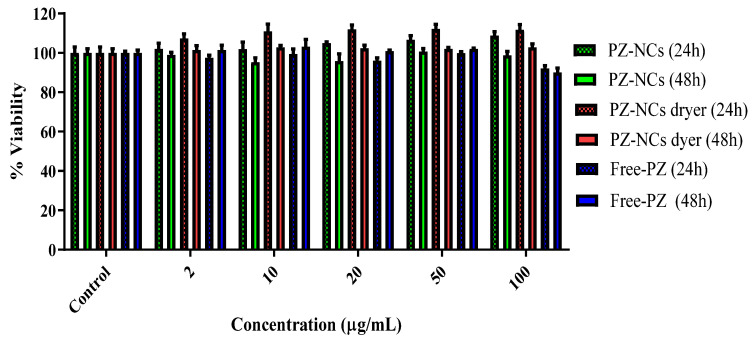
Evaluation of in vitro toxicity using the resazurin assay (alamarBlue™ assay). Variation of cell viability (%) with PZ concentration (µg/mL). Data are expressed as the mean ± SD (*n* = 6).

The extent of cell growth inhibition was measured using a Y-79 cell model at 24 h and 48 h with the alamarBlue™ assay. Different concentrations of PZ were either added in solution (Free-PZ) or encapsulated (PZ-NCs and PZ-NCs dryer) to the wells with the medium as a control in the Y-79 cell line. The cell viability after treatment with various concentrations of the different formulations is shown in Figure 4. The formulations were tested in the presence of polyvinyl alcohol (PVA), which is widely used as a stabilizer in nanoparticle synthesis [39] and cryoprotectant in freeze-drying processes [54].

It was observed that, even at the higher concentration (100 µg/mL) of PZ, the viability remained at 90% at 48 h. These data corroborate previous results, confirming that polymeric nanosystems are nontoxic in different cell models [51,55,56] and in ocular cell models [31,57].

### 2.6. SAXS Analysis

The tolerance and efficacy in vivo assays were performed with rabbits, which are one of the most commonly used species in ophthalmology-related studies for their ready availability, as well as their acute inflammatory response [58,59,60]. However, the study of the cornea ultrastructure with synchrotron X-ray diffraction was performed with pigs, which are used for surgery practices in the Faculty of Medicine of the University of Barcelona, for species correlation. The reason for using porcine eyes for this research is the similarity between pig and human eye morphology and tear film [61]. One of the most remarkable characteristics of the pig eye is the thickness of the cornea, which is around 1500 µm in the center, compared with that of humans, which measures around 500 µm.

**Figure 5:**
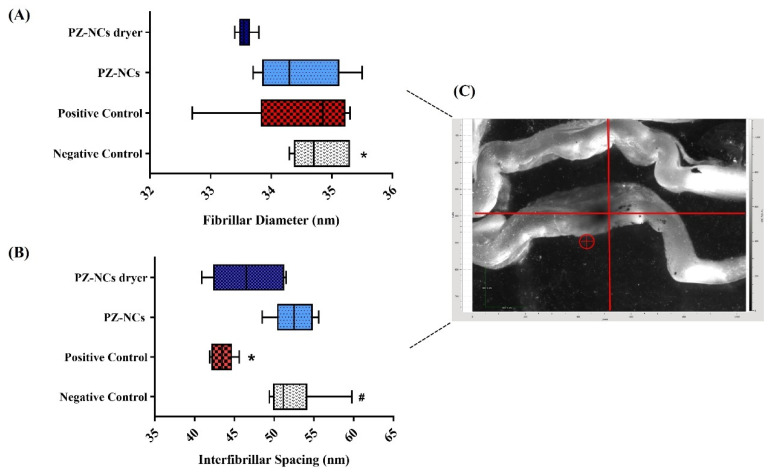
Series of data obtained from epithelium to endothelium in the center of the cornea (animal group, *n* = 4). Fibrillar diameter and interfibrillar spacing results using a box plot graph. Box: median, quartiles, and extreme values. Error bars: 95% confidence interval of the mean. (**A**) Fibrillar diameter; * *p* < 0.05 negative control vs. PZ-NCs dryer. (**B**) Interfibrillar spacing; * *p* < 0.05 positive control vs. PZ-NCs, ^#^ *p* < 0.05 positive control vs. negative control. (**C**) Image of the cornea positioned in the sample holder; the circle indicates the position of the beam.

Each cut of the cornea (four cuts per group) provided six scatter patterns from the anterior to the posterior part. For each position, the values of fibrillar diameter (FD) and interfibrillar spacing (IFS) were averaged; therefore, each value includes the variability between different animals. Table 1 and Table 2 summarize the fibrillar diameter and the interfibrillar distance, respectively. These values are represented graphically in Figure 5.

The results reveal a small decrease in the interfibrillar distance from the anterior to the posterior part of the cornea (Table 2), except for the group treated with PZ-NCs dried and resuspended (PZ-NCs dryer), as well as a more uniform fibril diameter across the explored data (Table 1). The results agree with the existing literature [62,63].

The ANOVA nonparametric test indicated that the differences found in fibrillar diameter were not significant between groups (*p* > 0.05) except for the negative control (no treatment) and treatment with PZ-NCs after spray-drying (*p* < 0.05) (Figure 5A). These results for fibril diameter agree with the literature data, indicating that this parameter is less affected by injury, inflammation, etc. [6]. As no significant difference was found between the positive and negative controls, the fibrillar diameter average was not appropriate to differentiate if the treatment with PZ-NCs damaged or recovered the cornea. The differences in the positions nearer to the endothelium were less significant between groups, which can be attributed to the fact that the inflammation was caused in the cornea periphery.

The interfibrillar spacing differences were significant when comparing the positive control versus the negative control, and the positive control versus treatment with PZ-NCs (*p* < 0.05) (Figure 5B). The large dispersion of the data obtained in the group of animals treated with PZ-NCs dryer could be the reason for no significant difference being found in this group versus the positive control group as was expected. The box plot in Figure 5B shows this phenomenon more clearly. This could be explained by the fact that, after damaging the cornea (via inflammation in our study), a first reaction was shrinkage, causing a decrease in the interfibrillar spacing (positive control). Moreover, when the PZ-NC formulation was instilled to avoid the inflammation, the interfibrillar spacing did not differ significantly between the negative control (no treatment) and the treatment groups PZ-NCs (*p* > 0.05) and PZ-NCs dryer (*p* > 0.05), thus providing a protective effect against inflammation. In addition, these results could indicate that fresh PZ-NCs recovered the corneal structure after the inflammation faster, but animals treated with stabilized PZ-NCs reacted more slowly (variability of values found). Therefore, it could not be concluded that the NC stabilization process succeeded in preserving the properties of the original formulation from the point of view of the SAXS results. The variability found in values of IFS of animals treated with dried NCs could be due to the slight increase in particle size and polydispersity of the NCs after the drying process. Future investigation related to stabilizing the PZ-NCs by freeze-drying should be carried out and evaluated, as the process is easier to control with respect to spray drying and is more widely used in the pharmaceutical industry [64,65]. Moreover, the number of animals used for SAX analysis should be increased to reduce variability, mainly in treated animals.

**Figure 6:**
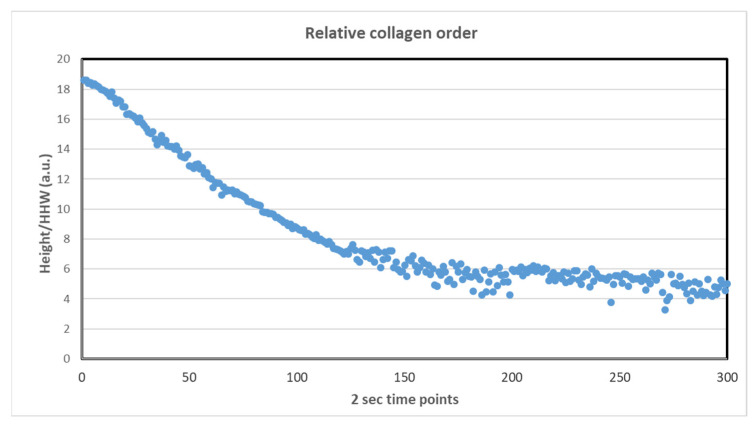
Height of the background-subtracted peak divided by peak width at half height (HHW) (arbitrary units, a.u.) versus the irradiation time (s).

The intensity of the first-order collagen scatters decreased with the irradiation exposure time, which confirms the damage caused by the irradiation (Figure 6). With continuous irradiation, the collagen structure was damaged over time, and diffraction patterns were weaker. This means that there was a disorder in the tissue. The intensity of the peak decreased from approximately 3.5 × 10^7^ to 2 × 10^7^ (from 0 s to 600 s, respectively) (Figure S2). However, during the first irradiations, there was no significant decrease in intensity, indicating that the degree of order in the fibrillar array did not change.

### 2.7. Corneal Transparency

**Figure 7:**
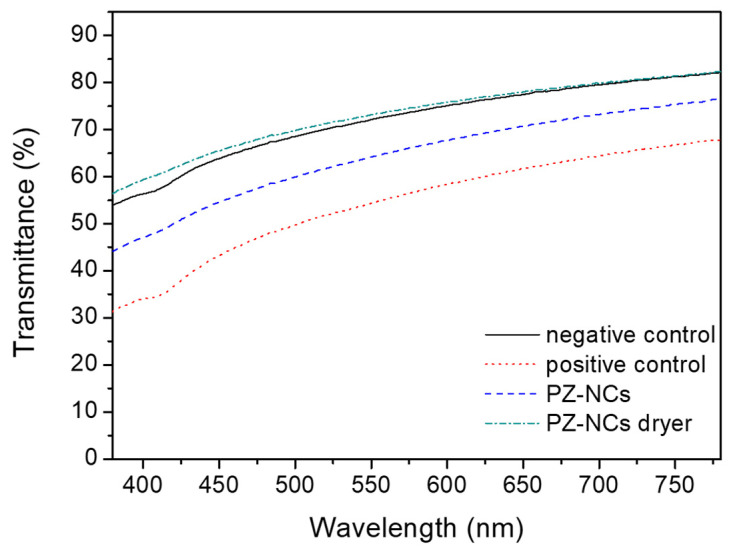
Transmittance values (%) as a function of wavelength in the visible region for the different studied pig corneas.

Loss of or reduction in corneal transparency could occur if the nano-based drug delivery systems damage the cornea [66]; therefore, light transmission was measured after ex vivo corneal formulation contact. In Figure 7, the absorbance values are shown as a function of the wavelength for differently treated corneas in the range of visible light. As expected, the positive control group showed lower transmittance values than the untreated cornea (negative control). On one hand, corneas exposed to PZ-NCs dryer almost recovered the clearness of the negative control, especially in the range of 500–780 nm. In addition, corneas exposed directly to the fresh PZ-NCs showed slightly lower values than the negative control. Transmittance experiments evidenced that the NC formulations did not alter the corneal transparency, and the lower transmittance values obtained with the fresh nanoparticle solution (PZ-NCs) could have been due to dispersion of light attributable to the presence of non-absorbed nanoparticles on the cornea surface, whereas PZ-NCs dryer fully penetrated the cornea. Further investigation can productively focus on how nanoparticle size affects the dispersion of light when intact absorbed nanoparticles remain on the cornea surface.

## 3. Materials and Methods

### 3.1. Materials

Diblock copolymer PLGA-PEG (Resomer^®^ Select 50:50 DLG mPEG 5000–5 wt.% PEG) was obtained from Evonik Corporation (Birmingham, AL, USA), Mw 95 kDa. The PZ was obtained from Capot Chemical (Hangzhou, China). Acetone, dimethylsulfoxide (DMSO), and polyvinyl alcohol (PVA) Mw 30–70 kDa, 87–90% hydrolyzed, were purchased from Sigma-Aldrich (Saint Louis, MO, USA). Reagents and chemicals for performing efficacy assays were purchased from Sigma-Aldrich and Thermo Scientific (Waltham, MA, USA). Milli-Q water (Millipore-Sigma, Saint Louis, MO, USA) was used for all experiments, and the reagents were of analytical grade.

### 3.2. Nanocarrier Synthesis and Stabilization Process

PZ-NCs were prepared using the nanoprecipitation technique first described by Fessi et al. [67]. The organic phase consisted of PLGA-PEG at a final concentration of 0.9 mg/mL and PZ at a final concentration of 1.0 mg/mL; beforehand, the drug was solubilized in 0.5 mL DMSO, and then mixed with the PLGA-PEG dissolved in 5 mL of acetone. This organic phase was added dropwise under magnetic stirring (700 rpm) into 10 mL of an aqueous solution of PVA 2.5% adjusted to pH = 4.5 with HCl 0.1 M. Then, acetone was evaporated under reduced pressure in a rotary evaporator Büchi B-480 (Büchi, Fawil, Switzerland). This formulation was optimized and characterized physiochemically beforehand [36].

Once the PZ-NCs were in suspension, the formulation was stabilized using a Nano Spray Dryer B-90 (Büchi Labortechnik AG, Fawil, Switzerland) to improve their long-term stability. Moreover, this instrument carries out the spray-drying process using a piezoelectric-driven ultrasonic atomizer to generate droplets, and the dried particles are collected using an electrostatic collector [68,69]. In our study, a spray mesh of 5.5 µm was used, the airflow rate was set to 140–150 L/min, and the spray rate was 75%; the outlet temperature was in the range of 25–30 °C [36].

The resuspension of the formulation was prepared by mixing the PZ-NC powder with Milli-Q water in a proportion of 1 mg/mL and vortex mixing for 1 min to ensure the deagglomeration procedure. Moreover, in our previous study both suspensions obtained were shown to be stable in the fridge for at least 6 weeks [36].

In all experiments, a comparative study was performed using both NCs (before and after the spray-drying process) to evaluate if the spray-drying process interfered with the drug efficacy. PZ-NCs were denoted for the initial formulation, pre-stabilization, and PZ-NCs dryer for the NCs after the spray-drying and resuspended process.

### 3.3. In Vitro Ocular Irritation Test (HET-CAM)

Fertilized hen’s eggs obtained from a farm (GALL S.A., Tarragona, Spain) were maintained at a temperature of 12 ± 1 °C for at least 24 h before placing them in the incubator with a controlled temperature (37.8 °C) and humidity (50–60%) for 9 days. Six chicken eggs were used for each formulation, and two eggs were used as controls (NaOH 0.1 M as positive control and NaCl 0.9% as negative control).

The shell was cut just above the marked line of the air cell. The inner membrane directly in contact with the CAM was moistened with 1 mL of 0.9% saline solution added with a pipette and incubated for a maximum of 30 min. Then, the saline solution was decanted, and the inner membrane was carefully removed using forceps, without causing injury to the blood vessels, to make visible the chorioallantoic membrane underneath. The test solutions (300 µL of PZ-NCs and PZ-NCs dryer) were then added directly into the CAM using a pipette and left in contact for 5 min.

Any vascular lysis, hemorrhage, and/or coagulation at different times, over a 5 min period after application of the test solution, was documented, and any effect was noted and compared with the controls: saline (negative) and sodium hydroxide (positive) solutions. The scores were recorded according to the scoring schemes described by Luepke [40] and the ocular irritation index (OII) was calculated using the expression formulated in INVITTOX protocol 1992 [70]. The following classification was used: OII ≤ 0.9 slightly irritating; 0.9 < OII ≤ 4.9 moderately irritating, 4.9 < OII ≤ 8.9 irritating, and 8.9 < OII ≤ 21 severely irritating.

### 3.4. In Vivo Draize Test

New Zealand white rabbits with no signs of abnormalities or ocular inflammation and weighing 1.8–2.2 kg were used. All experiments were performed in compliance with the standards of the ARVO (Association for Research in Vision and Ophthalmology) resolution for the use of animals in research, and the corresponding protocols were approved by the Ethics Committee for Animal Experimentation of the University of Barcelona.

Rabbits (*n* = 6/group) were used to assess the irritancy of the PZ-NCs by applying the methods described by Draize and Kay et al. [41,71]. A single instillation of 50 µL of the PZ-NCs and PZ-NCs dryer was placed in the conjunctival sac of the right eye, using an untreated contralateral eye as a control. The level of irritation was evaluated 1 h after application of the formulation, then after 1, 2, 3, 4, and 7 days. The analysis of ocular lesions in the cornea, iris (opacity), and conjunctiva (inflammation, congestion, chemosis, and discharge) was performed by applying the ocular irritation score by visual assessment of any changes in the cornea, conjunctiva, and iris [41,72].

### 3.5. In Vivo Efficacy Assay: Anti-Inflammatory and Histological Studies

To assess inflammation prevention, PZ-NCs was tested in rabbits (*n* = 3/group) from the animal facility of the Faculty of Pharmacy, protocol 326/19, approved 13 May 2021 (according to Catalan Government Decret 214/97 of 30 July), by the Animal Experimentation Ethics Committee (CEEA) of the University of Barcelona.

The anti-inflammatory efficacy of the PZ formulations was assessed using the method described by Spampinato et al. [73]. Firstly, 50 μL of a single dose of fresh PZ-NCs, PZ-NCs dryer, or 0.9% (*w*/*v*) isotonic saline solution (negative control) was instilled in the conjunctival sac of the right eye. The contralateral eye was used as an untreated control. After 30 min, ocular inflammation in the right eye was induced by administering 50 µL of sodium arachidonate (SA) 0.5% (*w*/*v*) dissolved in phosphate buffer solution (pH = 7.4). The inflammation was measured 30, 60, 90, 120, 150, and 180 min after the instillation of SA. The level of inflammation was quantified through ocular changes, which are shown as the sum of the inflammation score according to a modified Draize scoring system [72] expressed as the average ± SD of three replicates.

After the in vivo experiment, the animals were sacrificed by an intravenous overdose of sodium thiopental, and the eyes were collected and fixed overnight (ON) in 4% paraformaldehyde (PFA) in 20 mM phosphate-buffered saline (PBS), pH 7.4. Furthermore, samples were paraffin-embedded onto cassettes, and vertical histological sections were obtained, stained with hematoxylin and eosin, and mounted on microscope slides to be viewed at 400× with a Leica DMD 108 optical microscope.

### 3.6. Assessment of Cytotoxicity

The cytotoxicity of PZ-NCs and PZ-NCs dryer in comparison to the Free-PZ not encapsulated was determined in the Y-79 cell line (human retinoblastoma cell line; Cell Lines Service CLS, Eppelheim, Germany), exposed to different concentrations of PZ ranging from 2 μg/mL to 100 μg/mL, using the AlamarBlue™ assay (AB). AB (resazurin) is a sensitive oxidation–reduction metabolic indicator; after entering the cells and in the presence of metabolic reducing equivalent molecules (originating from cell metabolism), it fluoresces and changes coloration from blue to rose, thus leading to a shift in the absorbance spectrum.

The Y-79 cells were maintained in RPMI-1640 medium, supplemented with 10% (*v*/*v*) fetal bovine serum (FBS), 2 mM L-glutamine, and antibiotics (100 U/mL penicillin and 100 μg/mL of streptomycin), in an atmosphere of 5% CO_2_ in air at 37 °C. The cells were centrifuged, resuspended in a culture medium, counted, and seeded, after appropriate dilution, at 1 × 10^5^ cells/mL in poly-L-lysine pre-coated 96-well plates (100 μg/well) for adherence, which was achieved in about 24 h. After adherence, the culture medium was replaced by the test solutions.

The PZ-NCs, PZ-NCs dryer, and Free-PZ (firstly prepared by dissolving the drug in DMSO) were diluted with an FBS-free culture medium to achieve the desired final concentrations (test solutions) and then added to cells (100 μL/well). Microplates were placed in the incubator, and cells were exposed to test solutions for 24 h and 48 h. After the exposure time, the media containing the NCs, Free-PZ, and the control (culture medium) were removed and replaced by an FBS-free medium supplemented with 10% (*v*/*v*) AB and incubated. The absorbance readings were performed about 4–5 h after AB addition, at 570 nm (reduced form) and 620 nm (oxidized form) using a Multiskan EX microplate reader (MTX Lab Systems, Vienna, Austria; Virginia, VA, USA). Wells with culture medium without cells, containing 10% (*v*/*v*) AB, were used as negative controls, necessary for the calculation. Untreated cells were used as a control with 100% viability. The cell viability was calculated by the percentage of AB reduction, using equations as indicated by AB manufacturers and as described before [74].

### 3.7. Synchrotron Small Angle X-ray Diffraction (SAXS)

#### 3.7.1. Sample Preparation

Ocular specimens were obtained under veterinary supervision from pigs used in surgical University practices at the Faculty of Medicine, according to the Ethics Committee of Animals Experimentation at the University of Barcelona. Four groups of animals were obtained: (1) the negative control treated with saline solution 0.09% (*w*/*v*) (blank sample), (2) the positive control inflammation treated with sodium arachidonate (SA), (3) treatment with PZ-NCs, and (4) treatment with PZ-NCs dryer. Ocular inflammation was induced by administering 50 µL of SA 0.5% (*w*/*v*) dissolved in phosphate-buffered solution, pH 7.4. All samples of NCs treated groups were instilled after 30 min of the ocular inflammation. The animals were then sacrificed after 4 h of NCs application.

Pigs (male, weight 30–40 kg) were anesthetized with intramuscular administration of ketamine hydrochloride (3 mg/kg), xylazine (2.5 mg/kg), and midazolam (0.17 mg/kg). Once sedated, the propofol (3 mg/kg) was administered via the auricular vein, and, immediately afterward, they were intubated and maintained under anesthesia using inhaled isoflurane. After chirurgical experimentation, the animals were euthanized (250 mg/kg of sodium pentobarbital was administered through the auricular ear vein under deep anesthesia), and the eyes were immediately extracted and transported to the laboratory in dry ice. The whole corneas with a surrounding sclera rim were preserved in 4% formalin until they were used for the SAXS experiments (Figure S3).

#### 3.7.2. Data Collection

SAXS data were collected on the BL11-NCD-SWEET beamline at the ALBA Synchrotron Light Source (Cerdanyola del Vallès, Barcelona, Spain). Each pattern was generated from an X-ray beam with an energy of 12.4 keV and the sample to detector distance was 7.67 m. All small-angle X-ray scatter patterns were calibrated against AgBh (with a standard periodicity of 58.38 Å).

**Figure 8:**
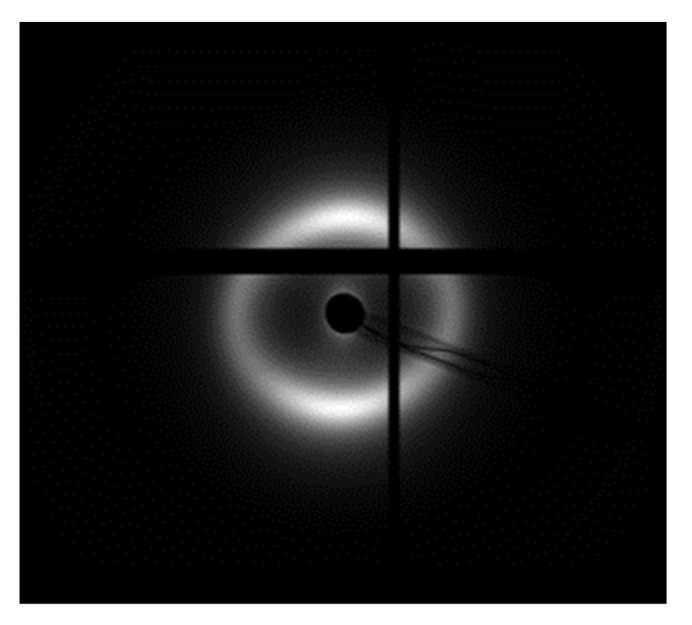
Example of a small-angle diffraction pattern from pig corneal stroma produced by collagen obtained in the Pilatus detector.

A thin strip of tissue, measuring approximately 1–2 mm wide, was dissected from the center of each cornea in the superior/inferior direction covering from limbus to limbus. After dissection, the corneas were wrapped in a plastic membrane to prevent dehydration, mounted in a sealed Perspex/Mylar chamber, and positioned ready for X-ray scattering data collection (Figure S4). The strips of swine corneas were positioned so that their cut edge was perpendicular to the incident beam direction, and SAXS patterns were obtained from the center of the corneas and from the anterior to the posterior part (Figure 8). Two experiments were performed.

Experiment 1. SAXS patterns were obtained at 250 µm intervals throughout the thickness of the tissue with an exposure time of 5 s, with four cornea cuts for each animal group. Six images per cornea cut were obtained covering the area between the epithelium to the endothelium.

Experiment 2 (radiation damage study). The cornea treated with PZ-NCs was irradiated 300 times at the same point with an exposure time of 2 s. The objective of the experiment was to study the effect of irradiation on the structure of the collagen during the experiments.

The data obtained (Table 1 and Table 2, and Figure 5) are averages from collagen fibrils of the different animals, along with the thickness of the cornea through which the X-rays pass; thus, they are highly representative of the tissue as a whole.

Data analysis for SAXS experiments was performed using the program SAXS4COLL [75,76], which was developed for the analysis of collagen-based tissues (Figure S5).

### 3.8. Corneal Transparency

Ex vivo swine corneas were obtained from pigs used in surgical practices at the Faculty of Medicine (University of Barcelona). The corneas were set out in the receptor compartment of Franz Cells with PBS (phosphate-buffered saline) in the chamber, and the temperature was kept at 32 °C. Four different corneas were treated with 1 mL of solution as follows: the positive control (using ethanol to damage the cornea), the negative control (saline solution), PZ-NCs, and PZ-NCs dryer. For 1.5 h, two drops of saline solution were added to each cornea every 10 min to simulate the natural clearing of the eye.

The transmittance through each sample was analyzed using a Nanodrop^®^ spectrophotometer TM 2000 (Thermo Fisher Scientific; Waltham, MA, USA). Briefly, samples were cleaned with saline solution to eliminate any contaminants and dried carefully under a N_2_ stream. Samples were mounted directly to the base of the instrument, and transmittance was measured in the range of 190–850 nm in at least three different regions of the cornea close to its center.

### 3.9. Statistics

Graphics and statistics were carried out using Windows-based programs: GraphPad Prism version 8 software package (GraphPad Software Inc., San Diego, CA, USA) and Excel Office 16 (Microsoft, Reading, Berkshire, UK).

Student’s unpaired *t*-test was used for two-group comparisons, and differences were taken as statistically significant when the *p*-value was below 0.05. All of the data are presented as the average ± SD.

For X-ray scattering, a one-way ANOVA test was performed between all groups, and, when significant differences were found, Dunn’s multiple-comparison test was used to analyze differences between pairs of groups, with the null hypothesis rejected at the 95% confidence interval.

## 4. Conclusions

In the current study, PZ-NCs were used as a nanotherapeutic system; they were developed to achieve their safe use in an ocular animal model before and after the spray-drying stabilization process. Moreover, with the administration of both formulations (PZ-NCs and PZ-NC dryer), it was possible to observe the effectiveness of the therapeutic prevention (significant decrease in the level of inflammation). In addition, the corneal structure was not affected, and the epithelium and cells were not altered by any NCs formulations.

Our study represents the first detailed description of the corneal ultrastructure (stroma) of pigs treated with PZ formulated in nanocarriers, analyzed by X-ray diffraction with synchrotron light, and the results demonstrated that the treatment did not appear to contribute to damage to or disorder of the cornea ultrastructure.

In summary, through different analyses and techniques, this study indicated that PZ-NC formulations did not cause any corneal damage within a 180 min time window of testing, and that these systems are safe and effective for the prevention of ocular inflammatory processes.

## Figures and Tables

**Table 1:** Fibrillar diameter (nm). Data are expressed as the mean ± SD (*n* = 4).

Position *	No Treatment	Positive Control	PZ-NCs Treatment	PZ-NCs Dryer Treatment
1	34.3 ± 0.5	35.3 ± 2.1	35.5 ± 1.8	33.5 ± 0.3
2	35.3 ± 0.3	35.0 ± 1.9	35.0 ± 1.3	33.4 ± 0.3
3	35.3 ± 0.3	35.2 ± 1.6	34.6 ± 0.8	33.6 ± 0.3
4	34.9 ± 0.1	34.7 ± 0.8	34.0 ± 0.3	33.6 ± 0.3
5	34.5 ± 0.3	34.2 ± 0.7	33.9 ± 0.7	33.8 ± 0.6
6	34.4 ± 1.2	32.7 ± 0.6	33.7 ± 1.0	33.5 ± 0.4

* Position: from 1 (epithelium) to 6 (endothelium).

**Table 2:** Interfibrillar spacing (nm). Data are expressed as the mean ± SD (*n* = 4).

Position *	No Treatment	Positive Control	PZ-NCs Treatment	PZ-NCs Dryer Treatment
1	59.8 ± 0.8	45.6 ± 9.9	53.5 ± 4.0	40.9 ± 9.8
2	52.3 ± 4.7	44.4 ± 8.1	55.6 ± 3.4	45.6 ± 10.4
3	51.0 ± 3.3	44.0 ± 7.9	54.6 ± 4.2	42.8 ± 10.9
4	49.4 ± 3.2	42.9 ± 6.1	51.5 ± 3.0	47.4 ± 11.1
5	50.0 ± 1.9	41.9 ± 5.5	51.0 ± 3.6	51.2 ± 19.1
6	51.3 ± 1.8	42.2 ± 5.7	48.5 ± 4.0	51.5 ± 16.3

* Position: from 1 (epithelium) to 6 (endothelium).

## Data Availability

Not applicable.

## Supplementary Materials

The supporting information can be downloaded at https://www.mdpi.com/article/10.3390/ijms231911184/s1.
