# Supplementary material for: Assessment of Efficacy and Safety Using PPAR-γ Agonist-Loaded Nanocarriers for Inflammatory Eye Diseases
Source: Int J Mol Sci. 2022 Sep 23;23(19):11184. doi: 10.3390/ijms231911184 (PMC9570464; doi:10.3390/ijms231911184)
Supplement: Supplementary file 1 [file ijms-23-11184-s001.zip › ijms-1872950-supplementary.pdf]

International Journal of *Molecular Sciences*

MDPI

Supplementary Materials:

Assessment of efficacy and safety using an Agonist PPAR-ɣ loaded nanocarriers for inflammatory eye diseases

AUTORS: **Esther Miralles [1], Christina S. Kamma-Lorger [2], Òscar Domènech [3,4], Lilian Sosa[5], Isidre Casals [1], Ana Cristina Calpena [3,4] and Marcelle Silva-Abreu [3,4]***

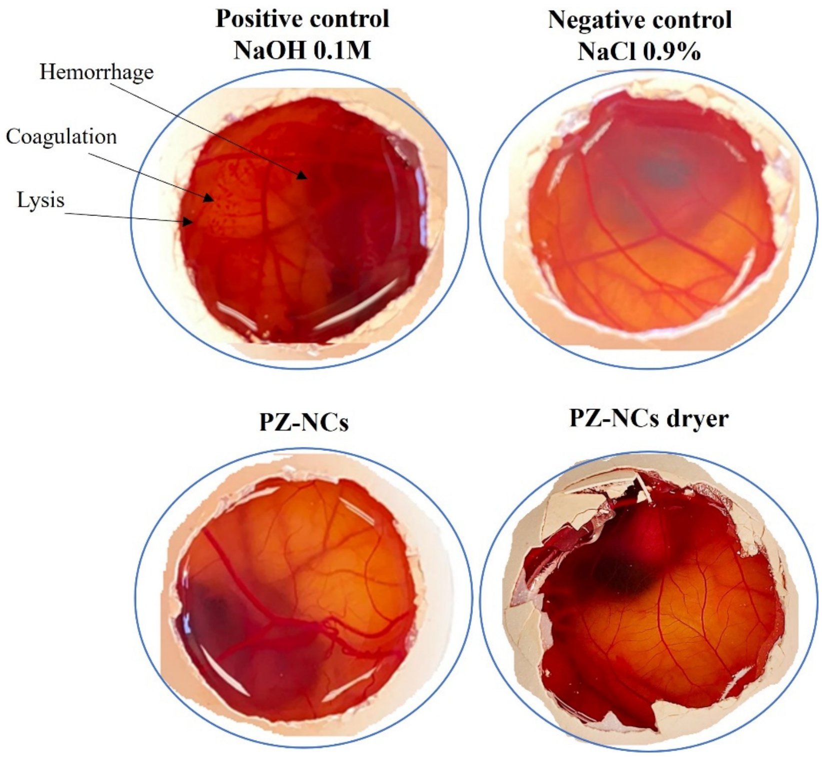

Figure S1. Photography of eggs used in the experiments of HET-CAM.

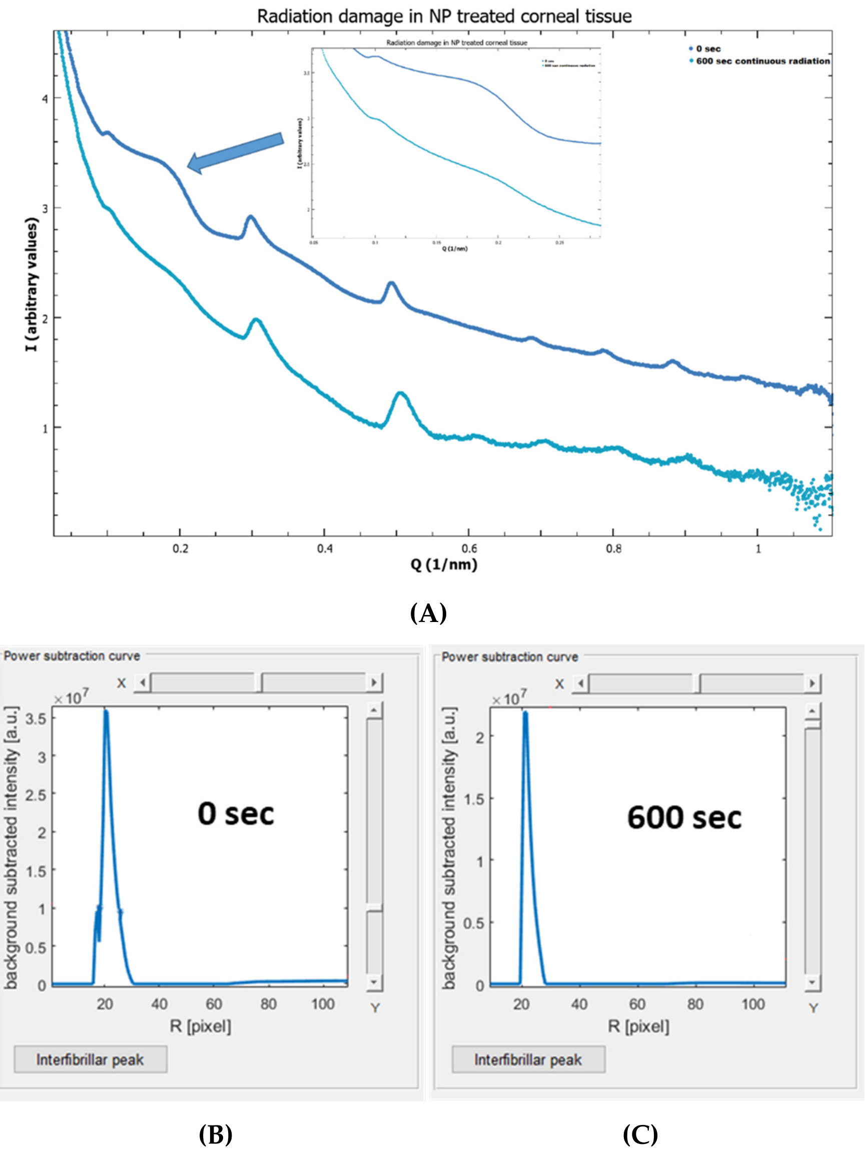

Figure S2. Radiation damage study in a cornea treated with PZ-NCs. (A) First-order collagen peak (B) Intensity of the peak at the first irradiation and (C) Intensity of the peak at the last irradiation.

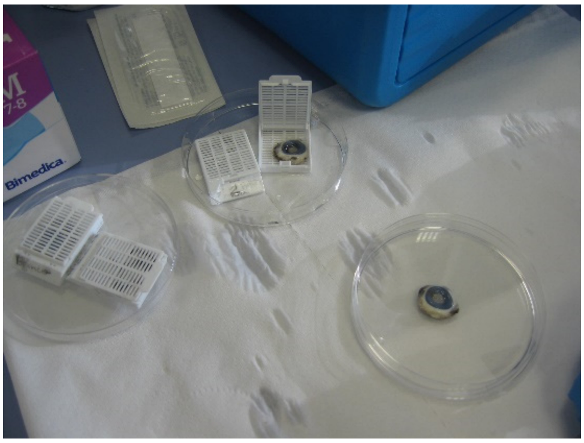

Figure S3. Photograph of pig eyes before separating the cornea.

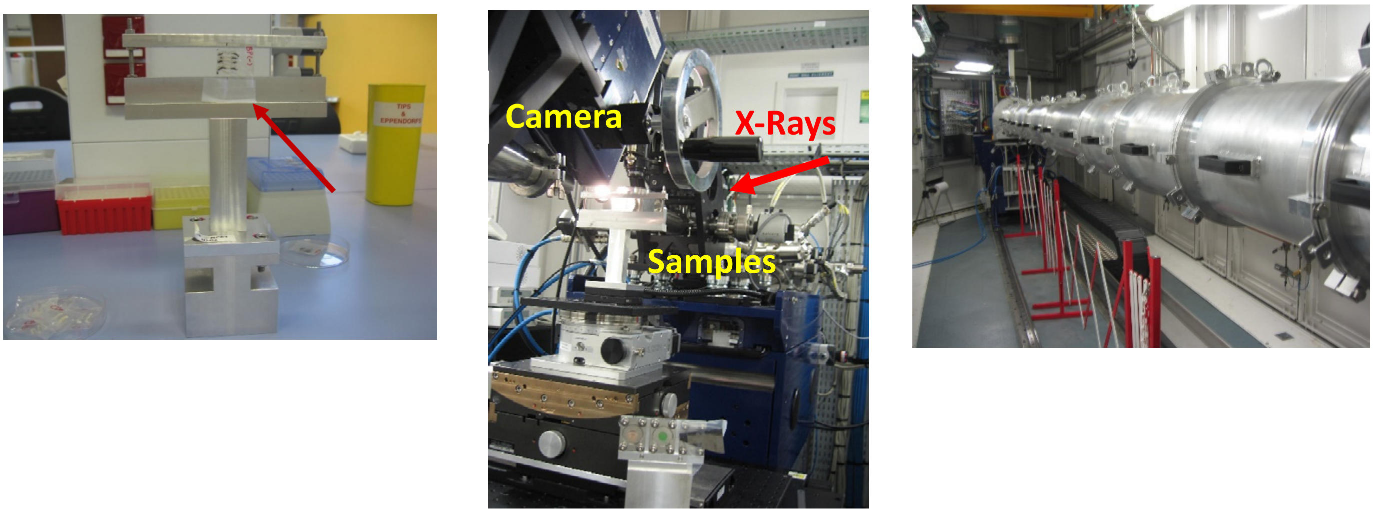

(A) **(B)** **(C)**

Figure S4. (A) Samples in the sample holder (the red arrow indicates the four corneal cuts); (B) Sample holder positioned in the beamline station; (C) Detector.

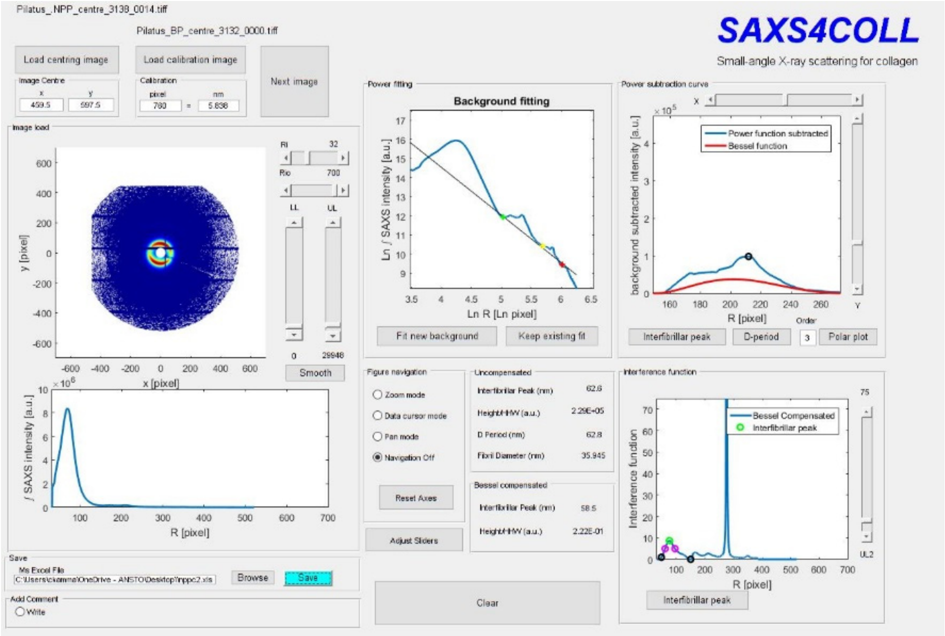

Figure S5. Illustration of SAX4COLL program when processing images (example of pig cornea treated with PZ-NCs SAXS pattern).
